# The Strong Antioxidant Sheep/Goat Whey Protein Protects Against mTOR Overactivation in Rats: A Mode of Action Mimicking Fasting

**DOI:** 10.3390/antiox8030071

**Published:** 2019-03-23

**Authors:** Efthalia Kerasioti, Aristidis Veskoukis, Christina Virgiliou, Georgios Theodoridis, Ioannis Taitzoglou, Dimitrios Kouretas

**Affiliations:** 1 Department of Biochemistry and Biotechnology, University of Thessaly, Viopolis, Mezourlo, 41500 Larissa, Greece; e-f-thalia@hotmail.com (E.K.); veskoukis@uth.gr (A.V.); 2 Laboratory of Analytical Chemistry, School of Chemistry, Aristotle University of Thessaloniki, 54124 Thessaloniki, Greece; cr_virgi@hotmail.com (C.V.); gtheodor@chem.auth.gr (G.T.); 3 Biomic_AUTh, Center for Interdisciplinary Research and Innovation (CIRI-AUTH), Balkan Center, 57001 Thessaloniki, Greece; 4 School of Veterinary Medicine, Aristotle University of Thessaloniki, 54124 Thessaloniki, Greece; jotai@vet.auth.gr

**Keywords:** sheep/goat whey protein, amino acids, mTOR, disease, fasting

## Abstract

Whey protein, a by-product of the cheese industry, can be putatively used as a functional food due to its beneficial health properties. The main objective of the present study was to assess in vivo the effect of a sheep/goat whey protein on the plasma amino acid profile and mammalian target of rapamycin (mTOR), a regulator of skeletal myogenesis. A control group was fed with a standard commercial diet while the experimental group received a standard commercial diet plus sheep/goat whey protein for 28 days. Liquid chromatography-tandem mass spectrometry (LC-MS/MS) was conducted to determine plasma amino acid levels while the expression of p70-S6 Kinase 1 (p70-S6K1) in liver and quadriceps muscles was quantified and used as a biomarker of mTOR activity. The results obtained showed a decrease in the levels of essential and branched-chain amino acids (BCAAs) in the experimental group. Furthermore, p70-S6K1 expression was decreased in the liver of rats consumed whey protein. In conclusion, the reduction of amino acid levels and the concomitant inactivation of mTOR imply that whey could potentially act protectively against disorders induced by mTOR overactivation. Intriguingly, this mode of action mimics fasting, an approach with established advantageous health effects.

## 1. Introduction

Proteins are fundamental biomolecules that are constructed by amino acids and perform a wide range of function, both from the technological [1,2] and nutritional [3] aspects. Indeed, they stimulate tissue growth and maintenance, they participate in numerous biochemical reactions, they act as messengers, they form immunoglobulins or antibodies, they transport and store nutrients and provide energy [4,5,6,7]. Proteins can be derived from different sources and might have either animal or plant origins. The idea that proteins derived from animals are richer sources of nutrients because they contain a greater arrow of amino acids, vitamins [8,9], metals [10,11] and fatty acids that are necessary for normal cell function compared to plant proteins used to be dominant. However, it appears that this is not the case [8]. Several experimental approaches have reported inconclusive results. Today we know that it is not an easy task to promote the notion regarding the superiority of foods rich in proteins of animal origin against the plant-derived protein products [12,13,14]. There is no compelling evidence backing up this superiority as indicated on the basis of cardiovascular and other pathologies [12,14]. Although, there are specific animal protein products that possess beneficial health properties. Whey belongs to this category.

Whey is a complex of proteins deriving from animal milk and has been characterized as a putative functional food with important health-related advantages. Milk, in general, constitutes a rich source of antioxidant compounds (e.g., tocopherols, retinol, carotenoids, ascorbate, phenols) including those that are derived from whey protein [15,16,17]. Niero et al. [18] determined for the first time the total antioxidant activity (TAA) of sheep, buffalo and goat milk. According to their findings, sheep milk showed the greatest TAA compared to buffalo and sheep milk and this could be attributed to its higher content in whey proteins, casein and fats. Recent evidence has demonstrated that sheep/goat whey protein as a food supplement possesses remarkable antioxidant properties at the molecular and tissue levels. Specifically, it has been found that sheep/goat whey protein possesses the ability to neutralize numerous free radicals, such as the commercially available 2,2-diphenyl-1-picrylhydrazyl (DPPH•) and 2,2’-azinobis(3-ethylbenzothiazoline-6-sulphonic acid) (ABTS•^+^) as well as OH• and O_2_•^−^ that normally exist in the organisms [19]. Furthermore, administration of sheep/goat whey protein in mouse myoblast C2C12 and endothelial Ea.hy926 cells exerted protective action against oxidative stress by enhancing the antioxidant defense and by confining protein and lipid peroxidation [19,20]. Additionally, sheep/goat whey protein improved the antioxidant defense of Ea.hy926 cells by activating antioxidant enzymes via the nuclear factor (erythroid-derived 2)-like 2/antioxidant response element (Nrf2/ARE) signaling pathway [21]. In another study, sheep/goat whey protein improved the redox status of blood and tissues in an in vivo model (i.e., rats) [22]. Furthermore, it improved the antioxidant ability of athletes and, hence, could potentially be used as a performance-enhancing substance [23,24]. Based on the above, it becomes evident that whey protein is a strong antioxidant that could be putatively used in order to protect against redox altering stimuli.

Whey proteins are considered high-quality proteins as they contain all the essential amino acids (EAAs) and, in fact, at higher concentrations compared to various plant protein sources, such as soy, maize, wheat and gluten [25]. The amino acids contained in whey are absorbed and used more efficiently compared to free amino acid solutions [26]. In addition, whey proteins contain higher concentrations of branched-chain amino acids (BCAAs) (with a content equal to 26% approx.), that is, leucine, isoleucine and valine, in relation to proteins originating from other sources [27]. All nine EAAs (i.e., phenylalanine, valine, threonine, tryptophan, methionine, leucine, isoleucine, lysine and histidine) cannot be synthesized de novo, therefore, they must be obtained through diet. They are involved in several vital processes with tissue repair, energy production, immune function and nutrient absorption among others. BCAAs also participate in protein and glucose homeostasis, body weight regulation and lipid metabolism [28,29,30,31]. In spite of their positive action, the excessive uptake of BCAAs can lead to differential pathological conditions as cardiovascular and neurological disorders [32,33,34]. It has been mentioned that plasma amino acid profile can be changed due to lipid accumulation and insulin resistance (IR) [35,36,37]. In particular, BCAAs levels have been found to be elevated in obese humans and animal models [38,39,40] and are implicated in IR, probably through the upregulation of the mammalian target of rapamycin (mTOR) complex 1 [41].

mTOR is a serine/threonine protein kinase that regulates diverse cellular processes, such as cell growth and proliferation, autophagy and protein synthesis [42,43]. It is established that there are two complexes of mTOR with distinct functions, the mTOR complex 1 (mTORC1) and 2 (mTORC2) [44,45,46]. While mTORC2 is implicated in cell proliferation and survival, mTORC1 is generally associated with cell growth and metabolism [47,48]. Specifically, it has been reported that mTORC1 promotes translation, transcription, lipid biosynthesis, whereas it inhibits autophagy [49,50,51]. mTORC1 regulates its function in response to various signals, such as growth factors, energy stress and amino acids [51,52]. Two well-known substrates of mTORC1 are p70-S6 Kinase 1 (p70-S6K1) and eukaryotic initiation factor 4E-binding protein 1 (4EBP1), which, when phosphorylated, enhance mRNA translation and, subsequently, protein synthesis [51]. The adaptor p62 is an important signaling molecule for the physiological function of mTORC1 since it interacts with signaling intermediates and activates substrate p70-S6K1 and 4EBP1 [53,54]. It is a molecule of utmost importance that interacts with the raptor, binds at the Rag proteins and contributes to their translocation in the lysosome [55]. Therefore, it seems to be a key regulator of mTOR activation. Taking into account the central role of mTORC1 on cell growth and metabolism, mTORC1 has been associated with pathological conditions like cancer, aging and metabolic diseases where growth and homeostasis are disrupted. The overactivation of mTORC1 by overfeeding may be a crucial factor for diabetes onset [51].

Based on the above, it becomes evident that whey protein as a substance rich in amino acids could exert its beneficial action by affecting the aforementioned molecular pathway. To our knowledge, there are no studies that approach the putative relation of whey protein with mTOR. Therefore, the objective of the present investigation was to examine the impact of sheep/goat whey protein on plasma amino acid levels in rats and to test the hypothesis that the pathway of mTOR activation is positively regulated in rat tissues. The obtained results could lead to the adoption of novel nutritional interventions alleviating pathologies that are related to mTOR.

## 2. Materials and Methods

### 2.1. Sheep/Goat Whey Protein Preparation

Sheep/goat whey protein was obtained from the Hellenic Protein S.A (Athens, Greece) and its content was 80 g in 100 g. It consists of β-lactoglobulin (47 g/100 g), α-lactalbumin (14 g/100 g), glycomacropeptide (13 g/100 g) and serum albumin (3 g/100 g). Its nutritional content consists of proteins (80 g/100 g), carbohydrates (10 g/100 g), fats (4 g/100 g), sodium (157 mg/100 g), potassium (397 mg/100 g), calcium (415 mg/100 g), phosphorus (319 mg/100 g) and magnesium (73 mg/100 g).

### 2.2. Experimental Animals

Twelve six-month-old male Wistar rats weighing 470 ± 30 g were used for this experiment, which was performed in the Veterinary Medicine School of Aristotle University of Thessaloniki in accordance to the Helsinki Declaration and National standards (Permission code EL54BIO10). The experimental protocol was approved by the National Veterinary Administration authorities on 13 July 2018 (Licence No.: 135973/851). The animals were housed in cages individually under controlled temperature (20–22 °C) and humidity (50–70%) and a 12-h light/dark cycle. Furthermore, they were acclimated for one week in the animal facility before the experiment took place. All animals were treated in accordance with the guiding principles of the European Community Council Directive (89/609/EEC) for the care and use of laboratory animals.

### 2.3. Study Design

**Figure 1:**
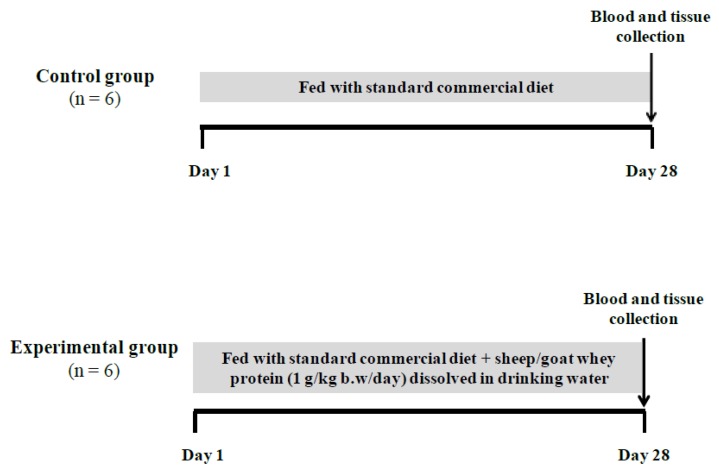
The study design. The control group was administered with the standard commercial diet; the experimental group was administered with the standard commercial diet plus the sheep/goat whey protein (1 g/kg of body weight/day) dissolved in drinking water. Animals maintained on their respective diet for 28 days. The downward arrows indicate the time of blood and tissue sampling.

The animals were randomly divided into two groups (i.e., 6 rats per group) as follows: the control group was fed with standard commercial diet containing corn, soybean meal, barley, bran, milk paste and molasses (purchased from Viozois S. A. Ioannina, Greece) and the experimental group was fed with standard commercial diet plus sheep/goat whey protein in a dose equal to 1 g/kg of body weight/day dissolved in drinking water. The exact daily volume of drinking water for each rat was determined and standardized by preliminary experiments in order to be sure that the rats consume the desired concentration of the control and experimental feed. The duration of the experiment was 28 days. Whey protein was selected because it contains higher amounts of BCAAs compared to other protein sources and, according to previous studies, high protein intake ameliorates IR [56,57,58,59] caused by disturbances in BCAAs catabolism due to attenuated branched-chain ketoacid dehydrogenase complex (BCKDC) activity [28,60]. The general health of the animals was observed daily. At the end of the treatment period, all animals were anesthetized with diethyl ether and blood samples were drawn by cardiac puncture. The animals died by exsanguination. Liver and quadriceps muscle tissues were also excised, snapped-frozen in liquid nitrogen and stored at −80 °C until further analysis. The study design is presented in Figure 1.

### 2.4. Metabolomics

#### 2.4.1. Reagents and Materials

LC/MS grade Acetonitrile (ACN), methanol (MeOH) were obtained from HiperSolv CHROMANORM (VWR, West Sussex, UK). Ammonium formate (NH_4_HCO_2_), ammonium acetate (NH_4_OAc) and formic acid (HCOOH) were purchased from Sigma Aldrich (Sigma Aldrich, GmbH, Taufkirchen, Germany). Distilled water (18.2 MΩ) for chromatographic separation was purified in a Milli-Q device (Millipore, Merch Darmstadt, Germany). The standards were of analytical or higher grade and for this study were obtained from various vendors. Anti-phospho-p70 S6 Kinase (Thr389) and anti-rabbit secondary antibody were purchased from Thermo Fisher Scientific (Invitrogen, New Delhi, India). The Glyceraldehyde 3-phosphate dehydrogenase (GAPDH) antibody was obtained from Sigma-Aldrich (St. Louis, MO, USA).

#### 2.4.2. Blood Preparation

Whole blood was collected in EDTA tubes, centrifuged immediately (1370× *g*, 10 min, 4 °C) and the supernatant (i.e., the plasma) was collected and stored at −80 °C. Prior to analysis, the samples were thawed and pretreated as follows: 150 μL of ice solvent mixture (ACN:H_2_O:MeOH, 70:15:15 *v*/*v*) was added to 50 μL of plasma and the mixture was vortexed for 5 min for protein precipitation. The samples were subsequently centrifuged (10,000× *g*, 15 min, 4 °C) and the supernatant was transferred into a vial for analysis. A pooled sample was prepared by mixing an equal volume of each of the test samples and was used as a quality control (QC) sample for the ultra-performance liquid chromatography-tandem mass spectrometry (UPLC-MS/MS) for targeted analysis.

#### 2.4.3. LC-MS/MS Analysis

For the metabolomic analysis, a previous reported hydrophilic interaction liquid chromatography-tandem mass spectrometry (HILIC MS/MS) method was applied and separation was performed on an Acquity- UPLC System (Waters Corporation, Millford, USA) by a BEH Amide column (2.1 mm × 150 μm, 1.7 μm) protected by an Acquity Van-Guard pre-column (Waters Ltd., Elstree, UK). The column temperature was maintained at 40 °C [61]. The mobile phase was a mixture of (A) ACN:H_2_O, 95:5 *v*/*v* and (B) H_2_O:ACN, 70:30 *v*/*v* both with a final ammonium formate buffer concentration of 10 mM. Elution was performed with a gradient program.

The MS/MS method monitors 101 multiple reaction monitoring (MRM) transition for the detection and quantitation of 101 hydrophilic metabolites comprising essential and non-essential amino acids, amines, organic acids and other small ionizable endogenous metabolites. All MS data were acquired on a XEVO TQD System (Waters Corporation, Milford, MA, USA) operating by the polarity switching mode. MS parameters were optimized for each individual analyte in terms of parent/daughter ion, dwell time, cone and collision energy (V). It has been shown that the method is sensitive, robust and efficient over a wide range of concentrations.

#### 2.4.4. Western Blot Analysis of p70-S6K1 (Thr389) Expression

The tissue samples were thawed and prepared as follows: 100 mg of tissue was homogenized with 500 μL of phosphate buffered saline [PBS (0.01 M, pH = 7.4)] and a cocktail of protease inhibitor tablet (Complete™ mini protease inhibitors, Roche, Basel, Switzerland) was added. The homogenate was vigorously vortexed and a brief sonication treatment on ice was applied. The homogenate was then centrifuged (10,000× *g*, 15 min, 4 °C) and the supernatant (i.e., the tissue lysate) was collected. In order to determine the expression levels of p70-S6K1, 40 μg of protein, as measured using the Bradford assay were used. Tissue lysates were separated by sodium dodecyl sulfate-polyacrylamide gel electrophoresis (SDS-PAGE) using an 8% polyacrylamide gel. Proteins were then transferred onto a polyvinylidene difluoride membrane (PVDF). The membranes were blocked overnight with 5% non-fat milk in a buffer (13 mM of Tris, 150 mM of NaCl, pH = 7.5) containing 0.2% Tween-20. Then, they were probed with a polyclonal rabbit anti-human/mouse/rat phospho-p70 S6 Kinase (Thr389) (1:600) primary antibody for 1 h at room temperature (RT). The membranes were then incubated with a polyclonal horseradish peroxidase conjugated goat anti-rabbit secondary antibody (1:5000) for 30 min at RT. The membranes were reprobed with a monoclonal anti-turkey/monkey/canine/chicken/human/bovine/rat/mouse/mink/rabbit/hamster glyceraldehyde 3-phosphate dehydrogenase (GAPDH) antibody (1:10,000) as an internal control. The optical density of the protein bands was measured by the Alpha View quantification software (Alpha Innotech, San Jose, CA, USA).

#### 2.4.5. Quantitation of Metabolites and Statistical Analysis

Data acquisition and the evaluation of chromatograms were performed by Waters MassLynx and TargetLynx version 4.1 (Micromass, Manchester, UK) software (SCN 882). The quantitative determination of endogenous compounds was performed by an external calibration; details on the method have been previously published [61]. All test samples, QCs and calibration standards were analyzed in one analytical batch. QC samples were analyzed every ten test samples. Calibration standards were analyzed in the beginning and the end of the analytical sequence as previously reported [51]. Multivariate statistical analysis was performed using the Simca-P v13.0 software (UMETRICS AB, Malmö, Sweden) and SPSS software version 21.0 (SPSS Inc., Chicago, IL, USA).

Orthogonal Partial Least Squared Discriminant Analysis (OPLS-DA) was used for modeling differences between the metabolic profiles of plasma samples from rats subjected to whey feeding conditions and for statistical evaluation of the models. Additionally, one-way ANOVA was applied in order to compare the means between the control and the whey protein feeding group. Differences were considered significant at *p* < 0.05 with the alpha level set at 0.025. All results are expressed as mean ± SEM.

## 3. Results

### 3.1. Effects of Sheep/Goat Whey Protein on Plasma Amino Acid Levels

**Figure 2:**
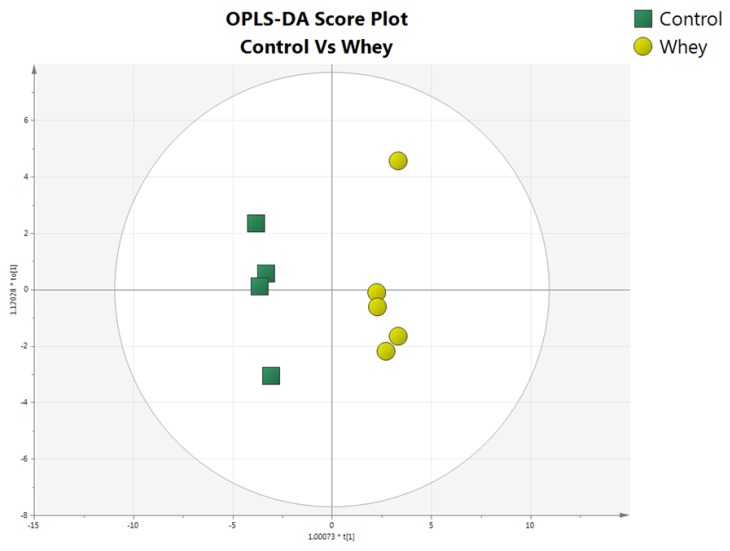
The Orthogonal Partial Least Squared Discriminant Analysis (OPLS-DA) score plot of plasma samples of whey protein fed rats against controls.

In total, 22 amino acids were detected in both the control and whey protein fed rats in the present study in order to evaluate the relationship between amino acid profile and mTOR activity after the administration of sheep/goat whey protein in vivo. Chromatographic peak areas were used for multivariate statistical analysis in order to find any differentiation between the studied groups. Discrimination of the sample groups was already revealed by principal component analysis (PCA) (data not shown), however, the partial least discriminant analysis was further performed to maximize the group differentiation and to identify potential biomarkers related to mTOR activity after different feeding conditions. The OPLS-DA provided a clear separation between plasma samples from control and whey protein fed rats. In Figure 2, 9 out of 12 samples can be seen due to the limited sample volume. The *p*-value of the Cross Validated ANOVA Analysis (CV Analysis) was 0.007, indicating the statistical significance of the investigated models. Additional statistical characteristics of the model R2Xcum and R2Ycum were 0.752 and 0.983, respectively, showing how well the model explains the variation in X and Y, respectively. The Q2Y that represents the quality and predictive power of the model was 0.95.

**Figure 3:**
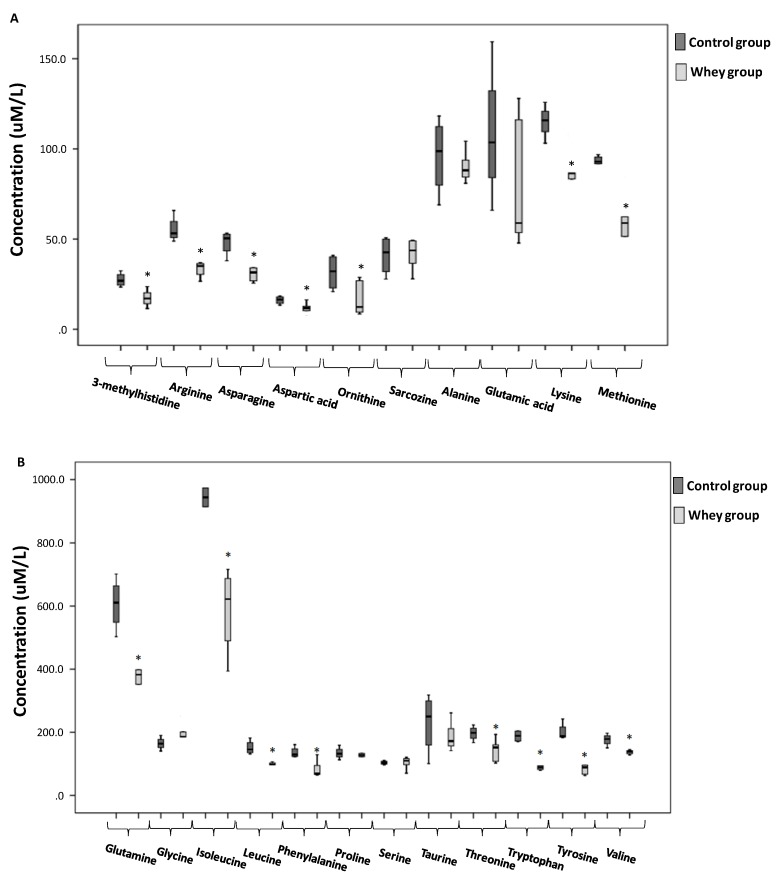
The boxplots illustrating the effects of sheep/goat whey protein administration on the plasma amino acid levels of rats. (**A**): 3-methylhistidine, arginine, asparagine, aspartic acid, ornithine, sarcosine, alanine, glutamic acid, lysine and methionine (**B**): glutamine, glycine, isoleucine, leucine, phenylalanine, proline, serine, taurine, threonine, tryptophan, tyrosine and valine. *: Statistically significant compared to the control group (*p* < 0.05).

Additionally, an unpaired student’s t-test was performed to establish and confirm the significance of important variables indicated by OPLS-DA. With regard to the amino acid contribution to that differentiation, reduced levels were observed for the majority of them in the whey protein fed group compared to the control. Specifically, 3-methylhistidine, arginine, asparagine, aspartic acid, glutamine, isoleucine, leucine, lysine, methionine, ornithine, phenylalanine, threonine, tryptophan, tyrosine and valine levels were reduced significantly by 37, 31, 33, 29, 37, 38, 34, 24, 35, 48, 40, 49, 45, 52 and 22%, respectively. On the contrary, no significant alterations were observed in the levels of alanine, glutamic acid, glycine, proline, sarcosine, serine and taurine between the two groups (Figure 3).

### 3.2. Effects of Sheep/Goat Whey Protein on Liver and Muscle p70-S6K Expression

**Figure 4:**
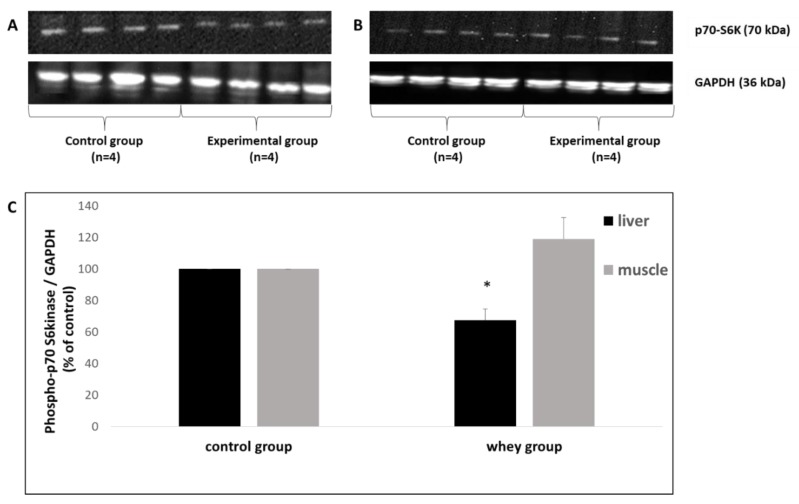
Representative western blots illustrating the effect of sheep/goat whey protein administration on the expression of p70-S6 Kinase (p70-S6K) in (**A**) liver and (**B**) quadriceps muscle of 4 rats. Glyceraldehyde 3-phosphate dehydrogenase (GAPDH) was used as the loading control for normalization. (**C**) The results are presented mathematically after the quantification through densitometry. *: Statistically significant compared to the control group (*p* < 0.05).

With respect to liver p70-S6K expression, it was reduced by 32.5% in the experimental group compared to the control group. On the contrary, no significant effect of sheep/goat whey protein on muscle p70-S6K expression was observed (Figure 4).

## 4. Discussion

This is the first study reporting a decrease in plasma amino acid levels after the administration of whey protein. Specifically, we report that sheep/goat whey protein administration in rats for 28 days led to decreased levels of 3-methylhistidine, arginine, asparagine, aspartic acid, glutamine, lysine, methionine, ornithine, phenylalanine, threonine, tryptophan, tyrosine and the BCAAs (i.e., isoleucine, leucine, valine). Moreover, sheep/goat whey protein decreased liver—but not muscle—p70-S6K (Thr389) expression. This is a substrate that is phosphorylated by activated-mTORC1, leading to translation and protein synthesis, whereas its extended phosphorylation induced by mTOR overactivation leads to several pathologies.

### 4.1. Characteristics of Sheep/Goat Whey Protein

Milk components have been recognized as functional foods suggesting that their use has direct and measurable health effects [62]. Milk proteins consist of approximately 20% whey protein and 80% caseins [63]. Whey protein has been characterized as a high-quality protein with many health benefits probably due to its content in bioactive substances, namely, β-lactoglobulin, α- lactalbumin, bovine serum albumin, lactoferrin, immunoglobulins, lactoperoxidase enzymes, glycomacropeptides and lactose [25,64,65]. A significant biological action of whey protein is its antioxidant activity as it enhances the concentration of antioxidant defense molecules and protects macromolecules from reactive species induced oxidative damage [19,20,21,23]. In comparison with other protein sources, whey protein contains higher amounts of BCAAs and especially leucine, which are important factors in tissue growth and repair. The number of studies in the literature examining the effects of whey protein on the plasma amino acid profile is limited and they mainly show that whey increases amino acid concentrations in plasma. Indeed, different forms of whey protein led to increased levels of both EAAs and BCAAs in rats [66] and diabetic mice [67]. The novelty of the present study is that whey protein decreased the amino acid profile in plasma. This is of remarkable biological interest as, according to other relevant studies, elevated plasma amino acid levels and particularly BCAAs and aromatic amino acid (AAA) levels have been linked to IR, type 2 diabetes mellitus (T2DM), cardiovascular diseases (CVD) and obesity [39,40,41,68] AAAs (i.e., phenylalanine, tryptophan, tyrosine) and BCAAs are considered biomarkers of metabolic disorders since it has been proposed that they predict T2DM and CVD for up to 12 years before disease onset and that they are correlated with metabolic syndrome (MS) diagnosis within 4 years [29,36,69]. Thus, nutritional intervention strategies based on sheep/goat whey protein appear to be a promising approach for the prevention of relevant disorders. A possible mechanism by which amino acids, and especially BCAAs, are implicated in insulin resistance (IR) might be the upregulation of mTORC1 through the phosphorylation of insulin receptor substrate 1 (IRS-1) [41].

### 4.2. mTOR Regulation by Amino Acids

Amino acids are important regulators of mTORC1 through Rag GTPases. When amino acids are present, Rag GTPases are converted into an active configuration and then interact with the raptor, promoting the clustering of mTORC1 on the lysosomal surface [70,71,72]. This localization allows mTORC1 to interact with the small GTPase Ras homolog enriched in brain (Rheb), which stimulates mTORC1 activity [70,73,74]. According to our results, p70-S6K1 (Thr389) expression was found decreased in the liver, whilst it remained unaltered in the skeletal muscle following whey protein administration. This finding indicates that mTORC1 is not overactivated and, thus, its harmful and disease-related action seems to be ameliorated due to the protective effect of whey protein. To this end, it has been reported that despite the positive role of mTORC1, its overactivation could lead to pathological conditions that are associated with growth and homeostasis dysregulation, namely, specific cancer types, metabolic disorders and aging [51]. mTORC1 regulates lipid accumulation and promotes the formation of white adipose tissue (WAT). The formation of WAT was found reduced in mice following an adipocyte-specific deletion of Raptor [75]. Furthermore, it has been shown that tuberous sclerosis complex 2 (TSC2)-deficient mouse embryo fibroblasts (MEFs) lead to increased mTORC1 activity and exhibited enhanced adipogenesis [76]. The overactivation of mTORC1 leads to lipogenesis in WAT, muscle and liver, which finally contributes to IR [51]. mTORC1 also over regulates fatty acid synthase, an enzyme that favors the rapid increase of cancer cells [77]. Being on the same page, the continuous activation of mTORC1 induces tumorigenesis by repressing autophagy, which normally exerts a tumor suppressive role [78,79,80]. Finally, mTORC1 inhibition may counterbalance the sources of cellular aging and activate repair mechanisms. To this end, it has been found that lifespan of yeasts, flies and worms was extended after mTORC1 inhibition [81,82,83,84]. This lifespan extension could be attributed to the enhanced autophagy following mTORC1 inhibition [85,86].

### 4.3. Effects of Sheep/Goat Whey Protein on Plasma Amino Acid Levels and mTOR Activation

**Figure 5:**
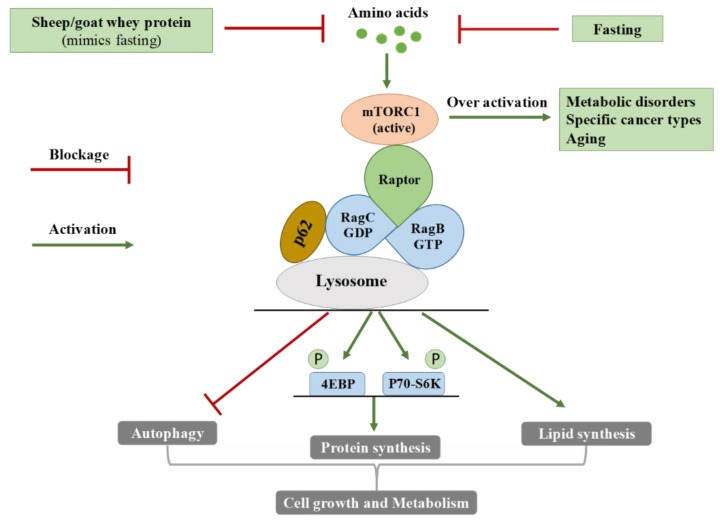
The molecular pathway of amino-acid-related (over) activation of mammalian target of rapamycin complex 1 (mTORC1) and the beneficial action of sheep/goat whey protein administration that mimics fasting. mTOR regulates diverse cellular processes, such as cell growth and proliferation, autophagy and protein synthesis and exists in two forms; mTORC1 and mTORC2. Two well-known substrates of mTORC1 are p70-S6 Kinase 1 (p70-S6K1), whose expression was measured in the present study and the eukaryotic initiation factor 4E-binding protein 1 (4EBP1). When they are phosphorylated, mRNA translation and, subsequently, protein synthesis are enhanced. The adaptor p62 is an important signaling molecule for the physiological function of mTORC1 since it interacts with signaling intermediates and activates S6K1 and 4EBP1. Whey protein, similar to fasting, inhibits the overactivation of mTOR and, therefore, exerts a potential protective role against diverse pathologies.

The present study describes the outstanding effect of whey protein along with its molecular background that has not been previously reported again. Intriguingly, it reduces the amino acid profile of plasma and it partially prevents the p70-S6K1 (Thr389) expression, thus leading to the putative inhibition of the adverse, disease-related effects induced by mTOR overactivation. This beneficial action of whey protein administration could be aptly parallelized with the mode of action of intermittent fasting (Figure 5). With respect to its definition, intermittent fasting refers to the eating regimen characterized by cycles of fasting and non-fasting during certain time periods. Recent scientific evidence supports the notion that during intermittent fasting, there is a metabolic switch leading to fatty acid mobilization and protection against several pathologies [87]. Interestingly, during fasting, plasma amino acids, as well as insulin levels are decreased [51,88]. The disturbance of the balance between food intake and energy needs increases the adenosine monophosphate/adenosine triphosphate (AMP/ATP) ratio, promoting the mTORC1 inhibition and autophagy activation [89]. Due to the above effects, fasting is applied for the attenuation or the symptom alleviation of T2DM, cardiovascular diseases, neurodegenerative disorders, aging and abnormal weight loss [90,91,92,93,94]. Furthermore, fasting delays the tumor onset, partly preventing cancer metastasis [95]. It acts protectively for normal cells against the adverse effects of chemotherapy [96], whereas it extends the lifespan in lower species, such as nematodes [97]. Therefore, our finding implies that sheep/goat whey protein could potentially be used for building eating habits with a similar pattern of action with fasting in order to help patients that suffer from disorders that mTORC1 overactivation is implicated in (Figure 5).

## 5. Conclusions

Amino acids are significant activators of mTORC1, an important factor that regulates cell growth and the metabolism. However, its overactivation leads to metabolic disorders, several cancer types and aging. Administration of sheep/goat whey protein in rats reduces amino acid levels in plasma and inactivates mTORC1 in the liver, therefore, it appears that it acts protectively. Specifically, the levels of 22 plasma amino acids were examined and 15 of them were reduced significantly in the experimental group compared to the control group. The reduction percentage ranged from 29% to 52%. Among them, the BCAAs (i.e., isoleucine, leucine, valine) whose elevated levels are implicated in IR probably through the upregulation of mTOR complex 1 are included. Furthermore, sheep/goat whey protein in liver reduced the p70-S6K1 (Thr389) expression by 32.5% in the experimental group compared to the control group, thus leading to the putative inhibition of the adverse, disease-related effects induced by mTOR overactivation. The mode of its action appears to mimic the beneficial impact of fasting on health. This is an intriguing piece of evidence indicating that sheep/goat whey protein could potentially be used as an alternative and promising nutritional intervention for the prevention or alleviation of diseases associated with homeostasis and growth disturbance.
